# Determining the Effect of Calculus, Hypocalcification, and Stain on Using Optical Coherence Tomography and Polarized Raman Spectroscopy for Detecting White Spot Lesions

**DOI:** 10.1155/2010/879252

**Published:** 2010-06-20

**Authors:** Amanda Huminicki, Cecilia Dong, Blaine Cleghorn, Michael Sowa, Mark Hewko, Lin-P'ing Choo-Smith

**Affiliations:** ^1^Faculty of Dentistry, University of Manitoba, 780 Bannatyne Avenue, Winnipeg, MB, Canada R3E 0W2; ^2^Department of Dental Clinic Sciences, Faculty of Dentistry, Dalhousie University, 5981 University Avenue, Halifax, NS, Canada B3H 3J5; ^3^Institute for Biodiagnostics, National Research Council Canada, 435 Ellice Avenue, Winnipeg, MB, Canada R3B 1Y6

## Abstract

Optical coherence tomography (OCT) and polarized Raman spectroscopy (PRS) have been shown as useful methods for distinguishing sound enamel from carious lesions *ex vivo*. However, factors in the oral environment such as calculus, hypocalcification, and stain could lead to false-positive results. OCT and PRS were used to investigate extracted human teeth clinically examined for sound enamel, white spot lesion (WSL), calculus, hypocalcification, and stain to determine whether these factors would confound WSL detection with these optical methods. Results indicate that OCT allowed differentiating caries from sound enamel, hypocalcification, and stain, with calculus deposits recognizable on OCT images. ANOVA and post-hoc unequal N HSD analyses to compare the mean Raman depolarization ratios from the various groups showed that the mean values were statistically significant at *P* < .05, except for several comparison pairs. With the current PRS analysis method, the mean depolarization ratios of stained enamel and caries are not significantly different due to the sloping background in the stained enamel spectra. Overall, calculus and hypocalcification are not confounding factors affecting WSL detection using OCT and PRS. Stain does not influence WSL detection with OCT. Improved PRS analysis methods are needed to differentiate carious from stained enamel.

## 1. Introduction

White spot lesions are areas of demineralized enamel that represent an early stage of caries, since they can progress to cavitated lesions if untreated [1]. These lesions appear chalky white when dried and are not visible radiographically. The demineralization process can be arrested or reversed by noninvasive means, including oral hygiene counselling and/or topical fluoride application [2, 3]. Therefore, early detection of white spot lesions (WSLs) is desirable since early preventive treatment can avert the need for future restorative treatment.

 New technologies such as the DIAGNOdent and quantitative light fluorescence (QLF) devices have been developed for the detection of early carious lesions. These techniques are intended to be adjuncts to clinical decision making and aid in planning preventive treatment [4]. Despite the potential of these methods, the results from these tools are affected by confounding factors in the oral environment, thereby compromising the sensitivity and/or specificity of these techniques. For example with the DIAGNOdent, stain, calculus, plaque [5], as well as developmental hypomineralization can produce a fluorescence output that results in false-positive readings [6]. A review of the literature evaluating the DIAGNOdent device found that, compared to visual assessment methods, the sensitivity was consistently higher but the specificity was lower, concluding that the increased risk of false-positives limits its clinical usefulness [7]. For QLF, stain, plaque, fluorosis, or any developmental hypocalcification results in false-positives [8–11]. Composite resins pit and fissure sealants as well as prophy pastes could also lead to false-positive results. Clearly, a new technology is needed that will not be affected by factors in the oral environment. A new optical approach to detect white spot lesions clinically is jointly being developed at the National Research Council of Canada-Institute for Biodiagnostics, the University of Manitoba and Dalhousie University, using a combination of optical coherence tomography (OCT) and polarized Raman spectroscopy (PRS). OCT is a nondestructive technique for high-resolution (10–20 *μ*m) depth imaging that gives information about the morphology and depth of white spot lesions up to 3 mm into enamel [12]. This method is similar to ultrasound technology, but instead of sound waves, light waves are utilized and the imaging is limited to near-surface tissues. Changes in the refractive indices of structures cause light backscattering, creating an image that is different for sound enamel and demineralized enamel. Previous studies have demonstrated the potential of OCT for caries assessment [13–16]. PRS is a noninvasive spectroscopic method that provides details on the biochemistry and molecular structure of white spot lesions. The energy difference between the incoming excitation light and scattered photons is proportional to the vibrational energy of molecules within the sample being studied, known as the Raman effect. The caries process results in biochemical and structural changes that can be followed by PRS, which uses scattered light to determine differences between the mineral matrix of sound enamel and demineralized enamel [16]. Our previous studies have shown that OCT and PRS can be used to distinguish sound enamel from white spot lesions (WSLs) *ex vivo* [14, 15, 17]. However, the effects of calculus, hypocalcification, and stain on OCT and PRS have yet to be established. By combining OCT and PRS, false-positive results from one technology can potentially be eliminated, thereby increasing the sensitivity and specificity of the new method. 

Calculus consists of an organic matrix with the inorganic components of dicalcium phosphate dehydrate (DCPD), hydroxyapatite (HA), octacalcium phosphate (OCP), and *β*-tricalcium phosphate (TCP). Raman spectra of human enamel are characterized by a main peak at ~959 cm^−1^ arising predominantly from the symmetric phosphate groups in carbonated hydroxyapatite, the major mineral component of dental enamel [18, 19]. Clinically, hypocalcification appears visually similar (chalky white) to a WSL and must be differentiated from a WSL since hypocalcification is a developmental defect that does not need to be treated. In hypocalcified enamel, the crystals are arranged normally but there are pores due to larger spaces between enamel rods [20]. Stain is often classified as intrinsic, extrinsic, or internalized [21]. Intrinsic stain occurs during tooth development due to alterations in the structure or thickness of enamel or dentin, extrinsic stain accumulates on the acquired pellicle, and internalized stain occurs when extrinsic stain is incorporated into areas of enamel defects after tooth development. 

The objective of this study was to determine whether calculus, hypocalcification, and stain are confounding factors affecting WSL detection with optical coherence tomography and polarized Raman spectroscopy.

## 2. Methods and Materials

**Figure 1:**
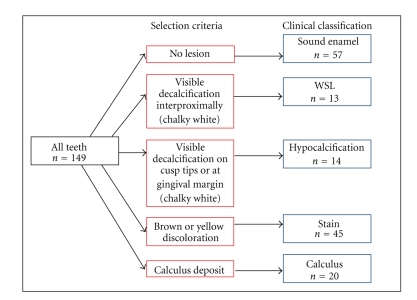
Schematic diagram outlining the sample sizes and allocation criteria for each of the 5 sample groups.

Teeth were obtained from consenting patient volunteers undergoing extractions for other reasons. Ethics approvals were obtained from the human ethics committees of the authors' institutions. Postextraction, the teeth were rinsed with water and clinically examined by two clinicians independently. Samples were separated into 5 groups: sound enamel, WSLs, calculus, hypocalcification, and stained enamel. Figure 1 is a diagrammatic representation of sample sizes and allocation criteria for the above groups. Since patient histories were not available, it cannot be determined if hypocalcified enamel was caused by excess ingestion of fluoride; therefore, this category is referred to as “hypocalcification”. 

**Figure 2:**
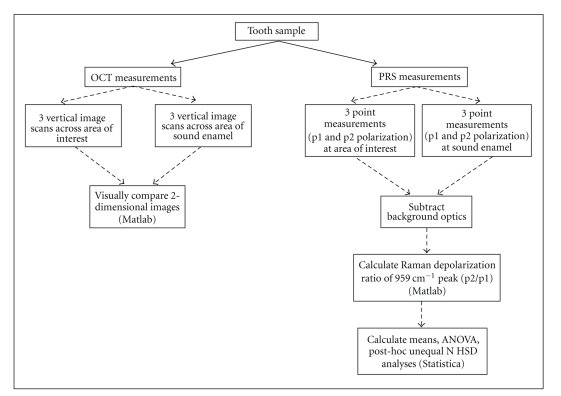
Flowchart of the OCT and PRS data collection and analysis methods.

Teeth were stored in deionized water prior to measurement with OCT and PRS. Figure 2 is a diagrammatic summary of data collection and analyses with OCT and PRS.

**Figure 3:**
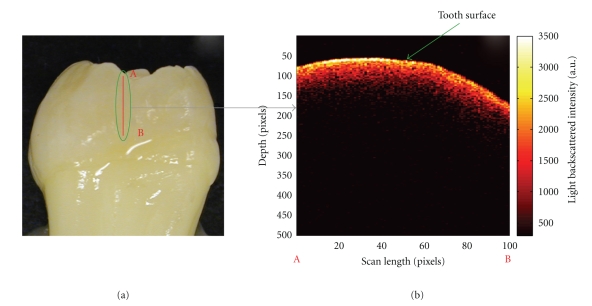
(a) Photo of an extracted human tooth with a line indicating the position of the OCT scan and (b) the corresponding OCT 2-dimensional depth image of sound enamel acquired from the laser line. Labels (A) and (B) indicate the two endpoints of the scan (a.u. = arbitrary units).

A Humphrey's system 2000 optical coherence tomography scanner (Zeiss Humphrey Systems, Dublin, CA, USA) operating at 850 nm was used for OCT measurements. For all data, the laser was focused to the thinnest line on the tooth surface and the scan length was 2.0 mm. Scans were acquired vertically along the incisal/occlusal to cervical direction. Three scans were collected across the area of interest with the proximal surface of interest oriented perpendicular to the laser beam. Three vertical scans were also taken of sound enamel on the same tooth surface for comparison. OCT images had display resolutions of 500 × 100 pixels, and transverse resolutions of 10–20 *μ*m. Figure 3(a) displays a photo of a tooth being scanned with the OCT system (laser scan line circled) with the corresponding 2-dimensional depth image of sound enamel (Figure 3(b)). MATLAB software (The MathWorks, Natick, MA, USA) was used to plot OCT images.

A LabRamHR Raman microspectrometer (HORIBA Jobin Yvon, Edison, NJ, USA) was used to acquire spectral data. The laser excitation was 830 nm and a 10x microscope objective was used (power at the sample was 125 mW) with an acquisition time of 5 seconds and 6 accumulations. On each surface, point measurements were taken with parallel- (p1) and cross- (p2) polarizations at a minimum of three different points. The optical configurations for parallel- and cross-polarization measurements have been described previously [17]. Background optics spectra were recorded and subtracted from each sample spectrum. Raman data were analyzed using MATLAB software to calculate the depolarization ratios (I_⊥_/I_∥_), where I_⊥_ is the area under the Raman band from 925–1000 cm^−1^ for cross-polarization (p2), and I_∥_ is the similar area for parallel-polarization (p1) [17]. This wavenumber range was used since it centres at 959 cm^−1^ (the main peak due to phosphate groups such as those found in apatite).

## 3. Results

**Figure 4:**
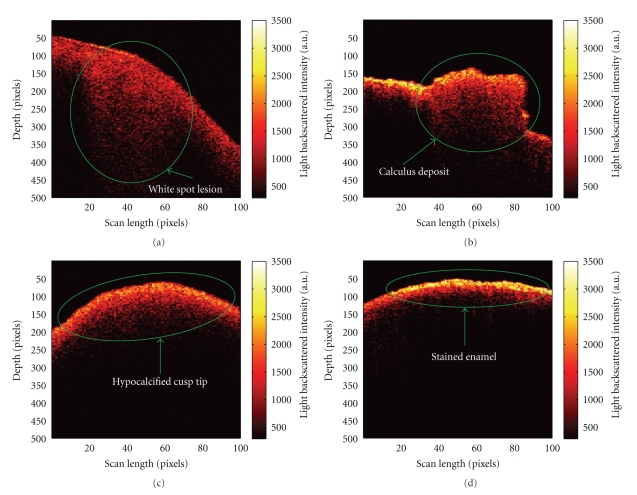
Representative OCT depth images of a (a) white spot lesion, (b) calculus deposit on a tooth surface, (c) hypocalcified cusp tip, and (d) region of stained enamel (a.u. = arbitrary units). All areas of interest are highlighted by the green markings.

Two-dimensional OCT images are shown of sound enamel (Figure 3(b)) and a WSL (Figure 4(a)). In the sound enamel image, there is intense light backscattering at the tooth-air interface, and no significant signal with depth into the enamel. In contrast, the OCT image of a WSL shows significant light backscattering beneath the surface with a triangular shape characteristic of a subsurface lesion as observed on histological sections. The two-dimensional OCT image displayed as Figure 4(b) shows a deposit of material on the tooth surface that is attributed to calculus. The OCT image of hypocalcification in Figure 4(c) shows diffuse light back-scattering and a more irregular subsurface pattern of scattering across the entire region scanned compared to sound enamel. The light back-scattering of hypocalcified regions is also more irregular than WSLs, which have a characteristic triangular shape. The OCT image of stained enamel in Figure 4(d) demonstrates increased light backscattering when compared to sound enamel, but again there is no triangular-shaped subsurface light back-scattering as observed with WSLs. 

**Figure 5:**
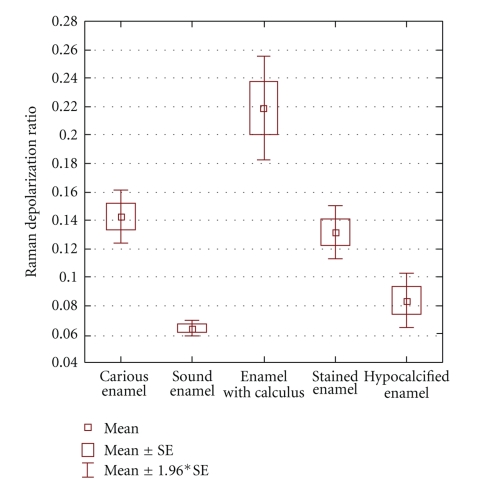
Box- and whisker- plot of the Raman depolarization ratios (mean, mean with standard error [SE], and mean with 1.96*SE) calculated from Raman spectra of regions of caries, sound enamel, enamel with calculus, stained enamel, and hypocalcified enamel.

**Figure 6:**
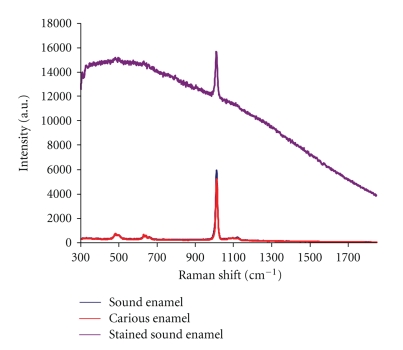
Representative parallel-polarized Raman spectra of unstained sound enamel, carious enamel, and stained sound enamel (a.u. = arbitrary units).

Average Raman depolarization ratios (*ρ*) from the various groups are depicted in a box-and-whisker plot (Figure 5). In order to determine whether the mean depolarization ratio values of the various groups were statistically significant from one another, a one-way analysis of variance (ANOVA) followed by unequal N HSD post-hoc comparisons (Statistica) was performed (Table 1). It was determined that the mean values were statistically significant in all cases at *P* < .05 except for three cases. Sound enamel was not statistically significant from hypocalcified enamel, carious enamel was not statistically significant from stained enamel, and lastly stained enamel was not statistically significant from hypocalcified enamel. In Raman spectra acquired from areas of stain (Figure 6), there is a large background sloping fluorescence that is not observed in spectra from areas of sound enamel or WSLs without stain where these spectra have a flat background. 

## 4. Discussion

The development of new technologies for WSL assessment requires that these devices undergo clinical validation prior to becoming an accepted clinical method. In addition to demonstrating the performance of these methods for providing high sensitivity for WSL detection, high specificity is also desirable. Optical coherence tomography and polarized Raman spectroscopy are methods that potentially can address the need for a technology with high sensitivity and high specificity. To date there are no studies outside our research group that investigate a combination of OCT and PRS to detect WSLs, and in particular the effects of the calculus, stain, or hypocalcification on the utility of these two technologies.

OCT imaging allows differentiation of sound from demineralized enamel on the basis of the characteristic triangular shape of the back-scattered signal beneath the tooth surface in images of WSLs. This subsurface scattering pattern is believed to be due to WSLs having porous enamel matrices, allowing incident light to travel further into the enamel and causing more scattering to occur, which is detected by the OCT system. 

When calculus is present, it appears clearly as a deposit in the two-dimensional OCT image. Similar to conventional caries assessment methods, scaling of calculus is recommended before an assessment is made using OCT and PRS. OCT can possibly be used to alert the clinician when calculus is still present in the region of interest and that further scaling is necessary. OCT images of hypocalcification can be differentiated from WSLs, since the light back-scattering found with hypocalcification is more irregular than WSLs and lacks the characteristic triangular shape. To some extent, hypocalcification can be differentiated from sound enamel with OCT since hypocalcification has a more irregular pattern of scattering at the surface and subsurface compared to sound enamel. Although areas of stain are not easily distinguishable from hypocalcified enamel or sound enamel with OCT, they can be readily differentiated from WSLs because areas of stain lack the subsurface triangular-shaped back-scattering pattern characteristic of WSLs. Further image analysis is required to nonsubjectively distinguish sound enamel from stained and hypocalcified enamel. 

Statistical analysis revealed that mean Raman depolarization ratios were statistically significant in all cases at *P* < .05 except for three cases: (a) sound enamel compared to hypocalcified enamel, (b) carious enamel compared to stained enamel, and (c) stained enamel from hypocalcified enamel. Since hypocalcified enamel can be mistaken for WSLs upon visual clinical examination, it is reassuring that the Raman depolarization values from hypocalcified enamel are distinct from caries, thereby increasing the specificity of the method. Based on the analysis focusing on the depolarization ratio of the 959 cm^−1^ peak, hypocalcified enamel could not be distinguished from sound enamel. This observation indicates that, fundamentally, hypocalcified enamel is like healthy sound enamel and not in need of treatment. In order to make this separation, further studies are needed which include examining the peak positions and peak width of the phosphate hydroxyapatite peak, which have been shown to be affected by the mineral crystallinity [22]. Furthermore, other peaks could be surveyed to look for peaks characteristic of specialized forms of hypocalcification such as fluorosis. 

The analyses indicate that stain complicates the use of PRS for discriminating stained sound enamel from carious enamel. In reviewing the Raman spectra acquired from areas of stain, it is observed that stained sound enamel spectra show a large background fluorescence that is not found in spectra from areas of sound enamel or WSLs without stain. In these preliminary analyses, a straight-line background in the region of the peak was simply subtracted for calculating the areas under the peak. Clearly, this initial approach is not enough as the curved background confounds this calculation. Further studies are consequently required using various algorithms to robustly fit the fluorescence background for elimination [23]. In addition, there are various instrument-based methods proposed to suppress the background fluorescence in Raman spectra [24]. With fluorescence-based devices (DIAGNOdent, QLF), stain chromophores from any source can lead to false-positive readings. With PRS, the fundamental basis of the method is the phosphate moieties specific to the dominant hydroxyapatite component from the mineral matrix. This peak itself is not due to staining and provides information on mineralization states as required for a method to detect demineralization in caries development. Therefore, with improved methods for fluorescence background subtraction/suppression, it is anticipated that staining will no longer confound PRS analyses.

The statistical analyses also indicated that, based on the Raman depolarization ratio, stained sound enamel cannot be distinguished from hypocalcified enamel. This is also not surprising since both groups are overall noncarious intact enamel with one group containing extrinsic staining. The underlying biochemistry of the enamel matrix is largely similar in both cases as revealed by the Raman spectra. Subsequent analyses such as those described above for examining spectra of hypocalcified enamel could provide insights for discriminating these two groups.

The presence of calculus leads to high Raman depolarization ratios. This result suggests that, like regions of demineralization, the apatite in areas of calculus has a disordered crystal structure and orientation as shown by higher depolarization ratio values. Calculus is easily observed on the OCT image, and therefore OCT will be the first technology used with the fibre optic probe to screen for WSLs and to determine whether PRS analysis of the lesion is necessary. If calculus is detected on the OCT image, the area will be scaled before the PRS method is applied to determine the Raman depolarization ratio. By combining OCT and PRS technologies, it is possible to rule out false-positive readings that might occur from using Raman depolarization ratios alone. Furthermore, according to the box- and whisker- plot (Figure 4), setting a depolarization ratio threshold of ~0.18 can help discriminate carious enamel from enamel with a calculus deposit.

It is important to note that this study was limited to extracted human teeth. In the oral environment, there will be a combination of many possible confounding factors present, and an additive effect may result in the oral cavity that was not observed when testing the possible confounding factors separately. These issues will be addressed in our subsequent studies as we transition to using fibre-optic-based devices for in vivo measurements with patient volunteers. 

In conclusion, calculus and hypocalcification are not major confounding factors affecting WSL detection using OCT and PRS. Stain does not influence WSL detection with OCT. With improved analysis methods, the current limitations with PRS analysis in the presence of stain will be overcome thus allowing better discrimination between carious enamel and stained enamel. The combination of OCT and PRS technologies can decrease the risk of false-positive reading and increase the potential for the detection of WSLs with high sensitivity and specificity. This initial study has pointed out limitations that should be taken into consideration when using these methods and highlighted further analyses that need to be undertaken to better understand the effects of calculus, hypocalcification, and stain on OCT and PRS technologies.

## Figures and Tables

**Table 1:** Results of ANOVA followed by unequal N HSD post-hoc comparison analyses with the *P* values shown. Mean Raman depolarization ratios (*ρ* *;* ± standard deviation) for each group are also displayed.

Group	Caries	Sound Enamel	Enamel with Calculus	Stained Enamel	Hypocalcified Enamel
Caries *ρ* = 0.14 ± 0.07	—	*P* < .001	*P* < .001	*P* = .97	*P* < .05
Sound Enamel *ρ* = 0.06 ± 0.04	*P* < .001	—	*P* < .001	*P* < .001	*P* = .84
Enamel with Calculus *ρ* = 0.22 ± 0.14	*P* < .001	*P* < .001	—	*P* < .001	*P* < .001
Stained Enamel *ρ* = 0.13 ± 0.11	*P* = .97	*P* < .001	*P* < .001	—	*P* = .09
Hypocalcified Enamel *ρ* = 0.08 ± 0.06	*P* < .05	*P* = .84	*P* < .001	*P* = .09	—
